# Motivations for continued tobacco smoking and reasons for quitting among youths in Wakiso district, Uganda: a qualitative study

**DOI:** 10.1186/s12875-023-02218-y

**Published:** 2023-12-05

**Authors:** Alex Daama, Stephen Mugamba, William Ddaaki, Grace Kigozi Nalwoga, Asani Kasango, Fred Nalugoda, Robert Bulamba, James Menya Nkale, Emmanuel Kyasanku, Ritah Bulamu, Gertrude Nakigozi, Godfrey Kigozi, Joseph Kagaayi, Stevens Kisaka

**Affiliations:** 1 Africa Medical and Behavioral Sciences Organization, Plot 7441, Nansana, Hoima Road, P.O Box 25974, Nansana, Uganda; 2 Makerere University School of Public Health, Kampala, Uganda

**Keywords:** Continued Tobacco Smoking, Youths, Motivations

## Abstract

**Background:**

Efforts have been invested towards cessation of tobacco use among youths aged 18–35 years, however, motivators for continued tobacco smoking and reasons for quitting are limited in Ugandan settings. Therefore, this study aimed to explore motivations for continued tobacco smoking and reasons for quitting in Wakiso district Uganda.

**Methods:**

This study used explanatory sequential method. Data from a Population-based survey collected from October 2019 to September 2020 was used to select participants for this qualitative study. Twenty-three in-depths interviews were conducted from July to October 2021 among youths (18-35years old) who reported continued tobacco use and those who quit. Data were analyzed using a team-based thematic content approach with the help of NVivo.

**Results:**

Data was collected from a total of twenty three participants, fourteen were tobacco quitters and nine were current tobacco smokers. Recurrent habit, desire to complement the use of other drugs, peer pressure, using smoking as a replacement for alcohol consumption, low tobacco prices, smoking as a tradition were reported as motivators for continued tobacco smoking. However, reported reasons for quitting smoking by youths included; packaging health warnings, school based prevention programs, fear of associated health risks due to tobacco use, embarrassment from family members.

**Conclusion:**

Targeted, and tailored tobacco prevention counselling through family support programs, intensified health education on the risks of smoking, and implementing stronger health warnings on tobacco packaging can be employed to reduce or stop tobacco use among urban youth.

## Introduction

Globally, tobacco use is considered the single most important cause of preventable morbidity and premature mortality worldwide [1]. According to WHO global report, about 80% of the 1.3 billion tobacco users worldwide live in low and middle-income countries [2]. Additionally, in Great Britain, the prevalence was estimated to be 16.9% in 2015 and was slightly lower in women than men [3]. In Australia, daily cigarette smoking has declined by 0.6% points per year over a similar time period (from 22.4% of adults aged 18 + years in 2001 to 14.5% in 2015) [4]. However, international comparisons are complicated since different nations have varying definitions of what constitutes a smoker. Different techniques are employed for calculating smoking prevalence for instance Australia only includes daily smokers in their headline statistics. Besides that, the headline prevalence rate for the United States is less than 16%, but this excludes occasional smokers, and the true proportion could be as high as 20% [3].

Low and middle income countries accounts for 80% of the smoking population [5]. In Africa, it is estimated that smoking prevalence will increase by nearly 39% by 2030, from 15.8% in 2010 to 21.9% by 2030 [6]. Recent trends in the region show an increase in tobacco use among girls and currently 13 million women smoke. The prevalence of tobacco use among girls, which ranges from 4.6 to 36.6%, has become as high as that for boys, which ranges from 7.8–36.5% [7]. The WHO has recommended that surveillance of the major risk factors for NCDs such as tobacco use is imperative to predict the future burden of NCDs. This intensifies efforts to identify interventions to reduce future burden and monitor emerging patterns and trends [8]. Several studies show that youth are more vulnerable to risky behavior like smoking [9–12]. Smoking at early age increases the risk of addiction [13], and hence this encourages regular smoking [14].Young people who smoke are at elevated risk of asthma, impaired lung function, effects on brain development [15].

In Uganda, tobacco use is a major risk factor for NCDs which accounts for 25% of all deaths in the country [16]. The prevalence of smoking among youth in Uganda has been reported to range from 11–16% [17]. Mpabulungi et al. also reported 21.9% prevalance of smoking among high school students in Arua [18], and 17.5% in Kampala [19]. Exploring motivations for continued smoking among youth is important especially in urban settings. Majority of the youth are unemployed, poor, and live in slums hence increasing the risk of non-communicable diseases (NCDs) [19]]. Studies have documented motivations for continued smoking as low costs of tobacco products, social norms, and accessibility to tobacco products and curiosity [20–22]. Besides that, health reasons, cost, knowledge about hazards of smoking, adverts for tobacco control have been suggested as the reasons to stop smoking [23–25]. However, motivations for continued smoking and reasons for quitting tobacco smoking may be different in the Ugandan context.

The Framework Convention on Tobacco Control of the World Health Organization requires parties to put into practice evidence based tobacco control measures, such as Article 8’s mandate to protect the public from tobacco smoke exposure. Article 13’s ban on tobacco advertising, promotion, and sponsorship (TAPS) [26]. In 2015, Uganda approved the Tobacco Control Act 2015, which outlaws all kinds of TAPS and the practice of smoking in all public places [27]. However, an assessment of practices related to protection of the public from tobacco smoke exposure reported 50% allowed smoking [27, 28]. In addition, different community-based organizations for example Uganda Youth Development Link (UYDEL) have implemented youth friendly interventions to reduce smoking and other drugs among youth [29]. Unfortunately, some youth continue to smoke at a rate that is deemed hazardous to their health. The rationale of this study is to provide research evidence to accelerate the country’s efforts to achieve the Sustainable Development Goal 3.4 [30]. Therefore, the aim of this study was to explore motivations for continued tobacco smoking and reasons for quitting among youths aged 18–35 years in Wakiso, Uganda.

## Methods

### Study design and participants

The study design was explanatory sequential. Potential participants from the AMBSO-Population Health Surveillance (APHS) dataset who reported to have ever smoked and stopped at the time of data collection (Survey round two) between November 2019 and October 2020. Those who reported to be currently smoking were approached and invited for the in-depth interviews through peer leaders and APHS community mobilizers. The information was obtained through the tobacco smoking modules of the semi-structured questionnaire administered as part of the AMBSO-PHS. The APHS aims have been previously described [31, 32].

Purposive sampling was employed to recruit participants for this study, with a focus on selecting individuals who had a history of tobacco smoking. The data collection period for this study took place from July to October 2021.

### Data collection

Data collection for this study involved conducting in-depth interviews with participants. The interviews were conducted in Luganda and translated into English, and each interview lasted between 30 and 60 min. All interviews were audio recorded for transcription purposes. Measures were taken to protect the privacy and anonymity of participants, and the interviews were conducted in a safe and secure place.

### Data Analysis

The qualitative data collected for this study were analyzed using thematic content analysis approach [33], with the assistance of a software program(NVivo). Transcripts were repeatedly read and coded by several co-authors. Textual codes were derived from recurrent topics and then themes were devised to form a comprehensive codebook [34]. In order to establish, refine, and categorize themes, an iterative process of data analysis was used. Co-authors identified and reviewed codes and categories, then looked into the variances, richness, and connections between them. Synthesis was supported by frequent team debrief meetings. Themes were produced using both inductive and deductive methods until data saturation was attained across participant sub-groups and topic guides [35]. A framework for data synthesis was used to enter emerging themes and categories.

## Results

### Baseline characteristics of participants

Demographic characteristics of the study participants are presented in Table 1. Twenty three participants were included in the study, 17 of whom were males. Among the smokers, seven were male and among those that had quit smoking, 10 were males. In terms of age distribution, majority of the participants (14) were 30–35 years (see Table 1).

**Table 1:** Baseline characteristics of in-depth interview participants

Variable	Current smokers	Quitters	Total
Sex			
Female Male **Total**	02 07 **09**	04 10 **14**	06 17 **23**
Age category			
18–24 years 25–29 years 30–35 years Total	01 03 05 **09**	01 04 09 **14**	02 07 14 **23**

### Results for motivations for continued Tobacco Smoking among current smokers aged 18–35 years

#### Recurrent habit/practice

The participants in this study reported experiencing an improvement in bodily sensations after initiating smoking behavior. This led to the development of a smoking tradition, which ultimately progressed into addiction. Participants described addiction to smoking as an intense physical sensation similar to a burning sensation within the body, which made it difficult to discontinue the habit. Furthermore, the study revealed that individuals who work at night are particularly susceptible to smoking addiction due to their tendency to smoke as a means of maintaining body warmth during cold nights. This, in turn, led to a reluctance to quit smoking, resulting in the behavior becoming entrenched as a habit.

*“I told you I sometimes choose to sleep when I feel the urge to smoke. Sometimes I cannot really sleep; I keep tossing on my bed”. ID02*.

*“every time you resist smoking, you feel like you are missing something in your life, that’s why at least smoke one cigarette just to feel that I have smoked then I feel okay”. ID06*.

*“I would not even focus on anything before smoking, I would feel like I have lost my sight, I felt like I was in jail but when I smoke even one cigarette, I would feel like my life was back to normal, that was the level of addiction I was at”. ID10*.

*“do you know using a wet cloth on your body when you feel fire inside you?” ID10*.

*“people that work at night, since its always very cold in the night, they say that they get warm after smoking since that smoke is always warm and it goes straight to the lungs” ID02.*

#### Complementing pre-existing habit/practice

Individuals who drink alcohol and those that use drugs such as Khat and Marijuana ultimately develop the desire to smoke tobacco either concurrently with these drugs or after using them. Gradually, they become addicted and later stopping the practice becomes a challenge. Further, participants mentioned that some individuals commonly opt for tobacco smoking when they run out of supply for the drugs they frequently use.

*“I only smoke one cigarette in the morning but if I have eaten mira, I smoke four cigarettes a day. I always really want to smoke cigarettes after I have taken mira. But if I am not taking mira, I usually don’t want to smoke cigarettes”. ID15.*

#### Desire to stop or reduce alcohol consumption

The study revealed that those who wanted to abstain from alcohol consumption adopted tobacco smoking as a substitute . These participants perceived alcohol consumption as more challenging to stop than tobacco smoking, and reported that smoking provided same pleasure as drinking alcohol . As a result, tobacco smoking became a more dominant behavior in these individuals’ lives, ultimately leading to a reduction or cessation of alcohol intake.

*“Every time I feel the thirst for drinking alcohol, I smoke a cigarette instead. Smoking a cigarette every time I feel a craving for alcohol, “anziza mumbera” [it brings my life back to normal]. …. I smoke to keep myself away from drinking alcohol during the day”.ID04*.

#### Affordability of Tobacco

The affordability of tobacco products, coupled with a strong sense of cohesion among tobacco smokers, often results in reciprocal exchanges within social networks in which individuals financially support one another in procuring these products, despite financial constraints. The practice of reciprocal exchanges within smoking social networks, is often motivated by the perception that such exchanges serve as investments in social capital.

*“if you learnhow to smoke, you learn how to share as well because today you may have, and tomorrow you don’t have but if you have and share, another time you don’t have, those who have will share with you and then you will call it a day, you haven’t slept hungry”. ID06*.

*“it is true that the low price of cigarettes is a motivator for continued smoking”. ID03*.

“*Okay, according to me, different sellers have different prices they tag to their products, for those that sell cheaply usually sell to a lot of people which brings about an increase in the number of smokers after all the tobacco is cheap for most people to afford in this case”. ID05*.

*“Yes, it is true because the more tobacco is sold at affordable prices, the more people will continue smoking but if the prices are hiked, the number of people who smoke will reduce because they will not be able to afford it. Cigarette is expensive not everyone can afford it daily especially when some are not working”. ID10*.

However, some can continue smoking even when the prices are high.

*“increasing the prices of tobacco will not affect use, an addict will buy cigarettes at any cost which cigarettes are readily available and can be accessed anywhere”. ID12*.

*“Even when the cost for a cigarette is high for example shs 1,000,(one thousand Uganda shillings) I would still afford to buy it to keep me away from being drunk during day. The price of a cigarettes does not affect me at all”. ID04*.

#### Smoking as a tradition

Some people cannot stop smoking tobacco because it is a practice they inherited from their parents and continue smoking during cultural practices.

*“I inherited this vice of smoking tobacco from my late grandparents, it is this way until now because I became an heir to my late grandfather who was a traditional healer I carried on the habit and I am now a traditional healer that smokes tobacco during my treatment processes with my patients”. ID05*.

*“it is in the clan or family for them to smoke” ID06*

#### Normalized familial smoking:

Some families normalized smoking as either an acceptable habit or a cultural norm.

“*A person living with relatives who smoke is likely to smoke. This is because it is within their “our”cultural (clan) practices/norm, we were initiated into tobacco smoking at early age, it is inevitable to smoke”. ID08*.

*“children grew up seeing everybody in the family smoking (both men and women) which inspired/motivated those who did not smoke to start/learn how to smoke to fit in society because almost everyone was smoking”. ID10*.

*“me am a munyarwanda man and I am the last born in my family, I grew up seeing my parents, grandparents and my siblings smoking. Therefore, I learnt this habit from them and this enabled me to fit in society because everyone around me was smoking. It made me feel like it was a norm smoke in our family since everyone was a smoker and I could not ask them why they were all smoking”. ID10*.

*“youth have continued smoking because they see it as a family/cultural norm for example during the period I smoked, all of us in the family were smoking and I thought that maybe it was a norm in our family/culture that you could inherit it from your ancestors and that you had to pass it on to the next generation”. ID10*.

*“I learnt how to smoke out of curiosity. I used to see my dad smoke more than a packet a day, so I tried it”. ID12*However, there are individuals who grew up in families where parents and relatives smoked but they did not initiate smoking. Some family members reject tobacco smoking based on fear associated with poor health. This is more common when they see their colleagues suffer with the effects of smoking.

*“that uncle of mine who died, I grew up with him, he was a typical smoker but never inspired me to smoke, in fact he was one of the reasons why I will never smoke again because he was badly affected, got tuberculosis, his lungs were affected and the doctors told us it was smoking that triggered the death of our uncle, so whoever choses to smoke, they do it for their own reasons but not parents”. ID14.*

#### Peer influence

The study reported that some people are influenced by their friends to continue smoking for instance participants revealed that one cannot fit into a group of friends who smoke unless they also smoke.

“*Peer groups. You cannot fit in a group of friends who smoke and yet you do not smoke. Some smoke cigarettes for adventure. However, it does not make sense not to smoke yet your group of friends does. So, it does not look good if you don’t do what they do”. IDI 015*.

*“Youth continue smoking because of peer influence or the people they associate with, if they are smokers, he/she will find themselves smoking. It becomes fun to them, it is like telling me to play football with my friends, I don’t find any problem with it, it even becomes a habit and may lead to addiction because some may reach a point whereby they feel like, they are missing something if they haven’t smoked in a day”IDI 014*.

*“Yes, very many times I see people here starting slowly and they eventually imitate what their peers do, with time you see someone starting to smoke and eventually they are smokers”. IDI 005*.

### Results for reasons for quitting Tobacco Smoking among former smokers 18–35 years

Some participants think that health messages from adverts that show dangers or health effects of smoking can help individuals to quit smoking. 

Health warnings on tobacco packaging *“I think youth stop smoking because they learn about the dangers of smoking when they see and listen to adverts or after seeing those that have been affected by smoking”. ID10_Stopped smoking*.

*“However, those advertisements did not have an impact on me personally. I learnt of the effects of tobacco smoking, and I decided to stop smoking tobacco”. ID10_Stopped smoking*.

#### School based prevention programs

This study revealed that school based prevention programs can help to keep young people tobacco free. The education program empowers youth with knowledge about dangers of smoking and they are likely to make informed decisions and hence quit smoking.

*“Yes, but now such things have reduced, even here in this community, some factors for example going to school, have helped to reduce/stop the actions of smoking because many get to know the dangers of smoking”. ID10_Stopped smoking*.

#### Fear of associated health risks

A participant mentioned that some youths considered quitting smoking after they experienced negative health effects of smoking, e.g., falling sick and being hospitalized.

*“One of the major reasons why the youths quit tobacco smoking, cigarettes or pipe is because some youth eventually fall sick from diseases such as tuberculosis and prolonged cough, therefore immediately they notice that smoking is the major cause for being hospitalized, they quit as a way of improving their health or keeping safe.”. ID05_Stopped smoking*

#### Embarrassment from family members

The participants in this study reported that confidentiality and being embarrassed by their family members lead them to stop smoking. Participants revealed that, if they continued smoking, they would either be punished or hated by their parents. Therefore, they believed that, quitting smoking would give them an opportunity to live in harmony with their family members.

*“Oh, my parents were very tough and would either punish you seriously or even hate you for life, so the only solution I had, was to stop the habit of smoking because doing something while hiding meant that I was doing something wrong thing and every time I had to smoke, I had to accompany it with a sweet or PK (chewing gum) to prevent the smell ”. ID02_ Stopped smoking*.

## Discussion

This study investigated the underlying motivations for continued smoking and reasons that led to smoking cessation among youth in selected communities of Wakiso district. Results revealed that developing a recurrent habit and tradition played a pivotal role in perpetuating tobacco use, with many individuals reporting feeling physically better while smoking and subsequently becoming addicted. Furthermore, participants reported peer pressure, and the desire to complement pre-existing habits as additional motivators for continued smoking. Additionally, some youths reported using smoking as a mechanism to reduce or stop alcohol consumption., Accessibility of tobacco products was also identified as a contributing factor. Overall, these findings provide insight into the complex and multifactorial nature of tobacco use among youth in the study population. Our findings were consistent with previous research by Krzysztof et al. who reported that youths continue to smoke in order to experience the pleasure, ultimately leading to addiction [36–38].

Our study also found that peer pressure is a significant motivator for youths to continue smoking, consistent with previous research in various settings [20, 39, 40] ]. This may be due to the tendency for individuals, particularly adolescents, to conform to group standards and behavior .

Furthermore, affordability of cigarettes has been identified as a motivator for youth smoking as demonstrated by previous studies [41–43] .

The primary reasons cited by participants for quitting smoking were the health warning labels on cigarette packaging, fear of associated health risks, and fear of familial embarrassment.

The participants reported that their decision to quit was driven by a desire to improve their health and address any ailments caused by smoking, as well as the fear of potentially contracting illnesses in the future. This aligns with the literature, which highlights health concerns as a primary factor in the decision to quit smoking [40, 44]. This is further supported by studies among former smokers, who reported that they quit to protect their health, and among current smokers, who cited as a primary motivation for cessation [36, 45].

These findings are consistent with those of a study conducted in Washington DC by Halpern MT et al., which identified health effects as a major motivator for quitting smoking [46, 47]. Therefore, intensified mass media health education campaigns on the negative health effects of smoking and smoking cessation may potentially encourage youth to quit tobacco use [48].

The qualitative results of our study were further supported by our quantitative findings, which revealed that majority of participants (eighteen) quit smoking due to the fear of getting sick, six participants were advised by their relatives and friends to stop smoking, only one participant reported having stopped smoking as a result of tobacco control campaign.

Our study also found that health warnings on tobacco packaging played a significant role in influencing individuals to quit smoking. This highlights the importance of well-designed health warnings and messages as an effective means of communicating health risks and increasing motivation to quit, which was consistent with previous research [36, 46]. We think implementing strong graphic/pictorial health warnings on tobacco packing are needed to reduce continued smoking [49].

 Our findings revealed that some smokers were encouraged to quit after feeling embarrassed when family members disapproved their smoking habit. This finding highlighted the importance of designing and implementing tobacco prevention cessation programs that include a family support system [50]. Additionally, our results also present a new finding that some youths use smoking as a mechanism to reduce or stop alcohol consumption.

This study has several strengths, including the use of highly qualified and experienced research assistants from Africa Medical & behavioral Sciences Organization (AMBSO) who possess expertise in data collection and probing techniques. These individuals are knowledgeable regarding longitudinal epidemiological research studies and have a deep understanding of smoking, which greatly enhanced the quality of collected data. 

This main limitation for this study was, despite interviews being done and recorded in Luganda, the interviews were then translated and transcribed into English, which may have led to errors in translation and the loss of cultural nuance, potentially affecting the depth and comprehension of participant responses. To address this, we had members of the study team and lecturers from Makerere University review all the data. This increased the reliability of the results by ensuring that the transcripts were read and remained within the cultural context.

## Conclusions

The findings revealed that motivators for continued smoking included recurrent smoking habit, complementing pre-existing habits, and desire to stop or reduce alcohol consumption, peer pressure, and curiosity. However, the desire to improve health, financial constraints, health warnings in tobacco packages, embarrassment from family members, fear of associated health risks, and support from family and friends were identified as reasons for quitting. The study highlights the importance of addressing the social and emotional factors that contribute to smoking behavior among youths in Wakiso district, as well as providing support for those who wish to quit.

### Recommendations

Targeted interventions such as tobacco prevention counselling sessions through family support programs, intensified mass media health education campaigns on the negative health effects of smoking and smoking cessation, the availability of resources for quitting, and implementing strong graphic/pictorial health warnings on tobacco packing are needed to reduce tobacco use and encourage quitting among the youth in the study area.

## Data Availability

The corresponding author is willing and ready to provide the de-identified datasets used and/or analyzed during the current study upon reasonable request.
